# Translation of Preclinical PET Imaging Findings: Challenges and Motion Correction to Overcome the Confounding Effect of Anesthetics

**DOI:** 10.3389/fmed.2021.753977

**Published:** 2021-10-22

**Authors:** Alan Miranda, Daniele Bertoglio, Sigrid Stroobants, Steven Staelens, Jeroen Verhaeghe

**Affiliations:** ^1^Molecular Imaging Center Antwerp, University of Antwerp, Antwerp, Belgium; ^2^University Hospital Antwerp, Antwerp, Belgium

**Keywords:** positron emission tomography, preclinical, brain, anesthesia, motion correction

## Abstract

Preclinical brain positron emission tomography (PET) in animals is performed using anesthesia to avoid movement during the PET scan. In contrast, brain PET scans in humans are typically performed in the awake subject. Anesthesia is therefore one of the principal limitations in the translation of preclinical brain PET to the clinic. This review summarizes the available literature supporting the confounding effect of anesthesia on several PET tracers for neuroscience in preclinical small animal scans. In a second part, we present the state-of-the-art methodologies to circumvent this limitation to increase the translational significance of preclinical research, with an emphasis on motion correction methods. Several motion tracking systems compatible with preclinical scanners have been developed, each one with its advantages and limitations. These systems and the novel experimental setups they can bring to preclinical brain PET research are reviewed here. While technical advances have been made in this field, and practical implementations have been demonstrated, the technique should become more readily available to research centers to allow for a wider adoption of the motion correction technique for brain research.

## Introduction

Positron emission tomography (PET) is a molecular imaging technique that allows to quantify the distribution of radiolabeled biomolecules in the living body. In the clinic, PET is commonly used for diagnostic workup and treatment monitoring in fields like oncology, neurology, or cardiology (1–4). In addition, PET is a valuable tool for clinical as well as fundamental research in these fields as it allows to investigate molecular mechanisms of several diseases or drugs and their efficacy.

Preclinical PET, commonly performed in non-human primates and rodents to help development and validation of novel radiotracers, investigate molecular mechanisms of disease in animal models, test drug safety, efficacy, and response to treatments. In contrast to PET in humans, animal scans are usually performed using anesthesia to maintain the animal still during the scan. Propofol, ketamine, and isoflurane are some of the most common anesthetics used for animal immobilization in preclinical PET (5). Unfortunately these compounds can have a pharmacological effect, affecting physiological parameters such as body temperature and cerebral blood flow, which in turn can affect the pharmacokinetics of a radiotracer (6). In addition, these anesthetics have been proven to work principally by interaction with neurotransmitter systems (7), which, for many neurological studies, are in fact the object of study. Because of these effects, the use of anesthesia in preclinical brain PET studies can be considered a confounding factor, and one of the principal limitations for translation of preclinical results to the clinic (8).

Several studies have investigated the effect of anesthesia on preclinical brain PET for many different anesthetics and tracers in the context of brain PET imaging. In the following sections these studies are summarized. In the second part we discuss motion correction solutions to circumvent the use of anesthesia in preclinical brain PET. Motion correction techniques offer the advantage of being potentially adaptable to common preclinical PET scanners, as well as allowing free animal motion.

## Anesthesia as a Confounding Factor in Preclinical Pet Studies

The first use of anesthetics dates back to the nineteenth century (7), however, despite being used for more than 100 years, it is only since the last decades that research on their molecular mechanisms of action has been possible thanks to the development of advanced techniques. Nonetheless, fundamental aspects of their mechanisms of action still need to be elucidated (9).

### Molecular Effects of General Anesthesia

Several hypotheses on the mechanisms of action of anesthetic agents have been formulated from the observation that the potency of anesthetic agents increased in proportion to its solubility in lipids. These theories stipulated that anesthetic agents changed the properties of the membrane lipid bilayer, such as its permeability, fluidity, and dimensions (10). Many of these theories were proved inconsistent with experimental results and were discarded. Although the focus has been shifted to the interaction of anesthetics with voltage and ligand gated ion-channels (see below), recent research has pointed again to the interaction of anesthetics with the cellular membrane, but instead of acting on the membrane bulk, it has been suggested that the interaction occurs at membrane rafts (11). Rafts are regions of ordered lipids (e.g., cholesterol, sphingolipids, and phospholipids) in the membrane, which in contrast to the more fluid bulk of the bilayer, are more rigid (12). Several ion channels associate with membrane drafts and it is believed that the interaction with these rafts can regulate the channel physiology (12). In a study by Pavel et al. (11), it is shown that anesthesia (e.g., chloroform and isoflurane) induced disruption of membrane rafts associated with the 2-pore domain K^+^ channel TREK-1, a mechano-, and thermo-sensitive K^+^ channel. This disruption activates this channel, which in turn affects anesthesia potency. This theory was further validated using TREK-1 knock-out mice. These mice displayed more resistance to anesthetics compared to control animals as measured by the onset time of anesthesia action, the loss of righting reflex, and the inspired minimum alveolar anesthetic concentration (11, 13).

As mentioned above, other mechanisms of action involve the direct interaction of anesthetics with voltage-gated and ligand-gated ion channels, the latter being the most frequent case (7, 9). For example, the anesthetic ketamine is an antagonist of the glutamatergic N-methyl-D-aspartate (NMDA) receptor, inhibiting glutamate binding to the receptor. However, it is not clear if blocking of the NMDA receptor is the main mechanism of action of anesthetics targeting this receptor (14). Using dizocilpine (MK 801), a more potent NMDA receptor antagonist than ketamine, no hypnotic effect is observed, suggesting that NMDA receptor antagonism is not the main mechanism of action of ketamine. Other receptors, such as nicotinic acetylcholine (nACh) receptors and cyclic nucleotide-gated potassium channels are other targets of ketamine that may play an important role in its anesthetic effect (14).

Another receptor targeted by anesthetic agents is the γ-aminobutyric acid type A (GABA_A_) receptor. Several anesthetics (such as propofol, isoflurane, and halothane) are known to interact with the GABA_A_ receptor (15) and potentiate its inhibitory effect. GABA_A_ is composed of several subunits such as α, β, and γ subunits. Using knock-out mouse models lacking one of these subunits, it has been found that some anesthetics act particularly on only some of these subunits. For example, in β_3_ knock-out mice the immobilization effect of the anesthetics enflurane and halothane was decreased (15), while the effect of isoflurane remained unchanged (16). The effect on the GABA_A_ receptor also depends on its location on the neuron. While synaptic GABA_A_ receptors respond to fast, transient inhibitory currents in response to presynaptic GABA release, extra-synaptic GABA_A_ receptors, located on the non-synaptic membrane, respond to low, ambient concentrations of GABA, producing a persistent inhibitory current (9). Interaction with extra-synaptic GABA_A_ receptors is thought to be responsible for the memory-related effects of anesthetic agents, since a low concentration of anesthetics can cause amnesia, but not immobilization. Moreover, anesthetics such as isoflurane and etomidate are attributed to increase cell surface expression of GABA_A_ receptors by change in extra-synaptic GABA_A_ receptor trafficking, effect which is associated with long-term cognitive changes (17).

Altogether, evidence suggest that anesthetics act by interacting with several voltage- and ligand-gated ion channels, and the different anesthetic effects (e.g., analgesia, hypnosis, and immobility) might be mediated by different receptors. Moreover, studies of gene expression changes caused by anesthetics (18) might give a better insight on their mechanisms of action. The reader is referred to reviews focusing on the molecular mechanisms of action of anesthetics for a more complete insight on the topic (7, 9, 10, 14, 15, 19).

### Effects of Anesthesia in Brain PET

The interaction of anesthetics on neuroreceptors and membrane permeability in the central and peripheral nervous system results in important physiological changes, including respiratory rate, cardiovascular function, and glucose metabolism (8). For instance, pentobarbital induces respiratory depression and reduced blood pressure in rodents (8). Ketamine administration causes muscle rigidity, and respiratory depression (20), while ketamine/xylazine reduces heart rate (21). Isoflurane depresses respiration without altering cardiac function (22), and increases cerebral blood flow (23). In addition, both ketamine and isoflurane induce hypothermia in small animals (24, 25). Changes in physiological parameters, such as cerebral blood flow and cardiac output, can in turn affect the biodistribution of PET tracers (8). In a review by Alstrup and Smith (6) the effect of several anesthetics on the brain PET reading is summarized.

Given that receptors with which anesthetic agents interact (e.g., dopamine and GABA_A_ receptors), can also be the target of PET ligands, anesthetics can modify the binding of these PET tracers. In this section we review several experiments that studied the effect of anesthesia on the brain PET reading of different tracers, summarizing the respective authors' hypothesis on the mechanism of interaction.

#### Methods to Study the Effect of Anesthesia in PET

As a first approach, the effects of anesthesia on the PET reading can be investigated by comparing PET scans performed using different anesthetics. This method allows visualization of the tracer kinetics from the onset of administration but does not reflect the awake state. To compare the state under anesthesia with the awake state, several approaches can be used: (i) Animals can be administered with the PET radiotracer in the awake state and, after some uptake period, sacrificed to perform autoradiography. Unlike dynamic PET, this method only delivers a single time point image of the radiotracer uptake in the sacrificed animal, but animals can be sacrificed at several time points to obtain pseudo-dynamic data. (ii) The animal can be administered with the radiotracer in the awake state, and following an awake uptake period (between 20 and 60 min uptake depending on the study), the animal can be anesthetized and scanned for the remainder of the uptake period. It is assumed that the effect of anesthesia is small and, if this is true, this scan reflects the awake state uptake. This approach does not allow to study the kinetics of the tracer from the onset of radiotracer administration, but pseudo-dynamic data can be obtained. (iii) The animal can be restrained during the scan and the tracer can be administered in the awake state. This method allows to perform the PET scan from the radiotracer administration onset in the awake state, but the stress caused by restraining can also affect the radiotracer uptake (e.g., due to activation/inactivation of stress related brain regions or neurotransmitters release, see section Effect of Physical Restrain in Brain PET Tomography). (iv) Using advanced methods, such as the specialized PET scanners or motion correction (described in section Small Animal Head Motion Tracking), the animal can be scanned in the awake state without physical restraining. These methods might be the optimal solution to visualize the tracer in the awake state, since no anesthesia or restrain stress is present. However, some stress might still be present by the scanning procedure itself and by restricting the animal motion to a reduced enclosure. In addition, administration of the tracer in the awake state might be challenging. Table 1 shows a summary of the studies presented in the next sections.

**Table 1:** Summary of the studies investigating the effect of anesthetic agents in PET brain radiotracers.

**Anesthetic(s)**	**Method**	**Outcome**
**[^18^F]FDG (glucose metabolism)**		
Ketamine + xylazine, ketamine, chloral hydrate, pentobarbital, propofol, and isoflurane	Autoradiography	Decrease glucose metabolism under anesthesia compared to awake (26).
Isoflurane	Awake uptake	Difference between saline and morphine withdrawal observed in anesthetized but not awake rats (27).
Isoflurane, α-chloralose	Anesthetics comparison	Isoflurane: increased FDG uptake after caffeine challenge, α-chloralose: decreased FDG uptake after caffeine challenge (28).
Isoflurane	Restrained scan	Reduced uptake and faster tracer kinetics in anesthetized compared to awake (29).
MMB, ketamine + xylazine, chloral-hydrate, pentobarbital, propofol, and isoflurane	Restrained scan	Decreased two-tissue k_3_, and regional increased or decreased cerebral blood flow, in anesthetized compared to awake (30).
Isoflurane	Awake unrestrained	Faster tracer kinetics and lower brain SUV in anesthetized compared to awake (31).
Isoflurane	Awake unrestrained	Decaying brain FDG uptake after awake uptake period in anesthetized mice, but not in awake mice (32).
**[^11^C]SCH23390 (dopamine D^1^ receptor)**		
Chloral hydrate, ketamine, and pentobarbital anesthesia	Restrained scan	Higher binding potential in chloral hydrate and ketamine, lower in pentobarbital, compared to awake (33).
**[^11^C]raclopride (dopamine D^2^ receptor)**		
Isoflurane, fentanyl-fluanisone-midazolam	Anesthetics comparison	Doubled binding potential in isoflurane compared to fentanyl-fluanisone-midazolam (34).
Isoflurane	Restrained scan	Lower binding potential in whole-body restrained and anesthetized mice compared to free-walking restrained (35).
Ketamine-xylazine	Awake unrestrained	Reduced binding potential in anesthetized rats (36).
**[^11^C]-(+)-PHNO (dopamine D^2/3^ receptor)**		
Isoflurane	Autoradiography	Greater increase in binding potential after amphetamine challenge visible in anesthetized rats but not in awake rats (37).
**[^11^C]cocaine (Dopamine transporter blocker**)		
Isoflurane, α-chloralose	Anesthetics comparison	Clearance of [^11^C]cocaine from the brain was faster in isoflurane-anesthetized rats than in α-chloralose rats (38).
**[^18^F]FPWAY (Serotonin receptor 5-HT^1A^)**		
Isoflurane	Awake uptake	Higher distribution ratio than conscious (39).
**[^18^F]MK-9470 (Type 1 cannabinoid receptor)**		
Isoflurane and pentobarbital	Awake uptake	Higher or lower regional relative SUV compared to conscious (40).
**[^18^F]flumazenil (GABA_A_ receptor)**		
Isoflurane, ketamine/dexmedetomidine	Awake uptake	Frontal cortex and hippocampus uptake in isoflurane and ket/dex anesthetized mice was 10 and 3-fold higher than in awake mice, respectively (41).
**[^11^C]-(R)-Rolipram (Phosphodiesterase subtype 4)**		
Isoflurane	Restrained scan	Tracer B_max_ and K_D_ significantly higher in conscious compared to anesthetized (42).

*MMB, medetomidine, midazolam, and butorphanol; Ket/dex, ketamine/dexmedetomidine*.

#### Comparison of Different Anesthetics

Using [^11^C]raclopride, a dopamine D_2_ receptor antagonist, striatum binding potential was doubled when using fentanyl-fluanisone-midazolam compared to isoflurane anesthesia in rats scans (34). Authors point out that different baseline binding potential depending on the anesthetic used might cause differing binding potential changes in challenge experiments.

The anesthetics isoflurane and α-chloralose were compared in the uptake of [^11^C]cocaine (dopamine transporter antagonist) in the rat brain (38). This tracer was used in a cocaine challenge to study the physiological response to cocaine under different anesthetics. Using laser Doppler flowmetry and in parallel with multi-wavelength optical spectroscopy, cerebral blood flow, cerebral blood volume and tissue hemoglobin oxygenation was measured. These parameters were decreased in rats anesthetized with isoflurane compared to rats anesthetized with α-chloralose. Moreover, the clearance of [^11^C]cocaine from the brain was faster in isoflurane-anesthetized rats than in α-chloralose rats. Different interaction of cocaine with the anesthetics, e.g., due to increase in intracellular calcium caused by cocaine, might have caused these differences (38).

Brain glucose metabolism has been investigated in a caffeine challenge, comparing the [^18^F]FDG uptake in isoflurane and α-chloralose anesthetized rats for different caffeine doses (28). While in isoflurane anesthetized rats the highest caffeine dose significantly increased tracer uptake in several brain regions compared to baseline, the opposite effect was observed in α-chloralose anesthetized rats, i.e., lower tracer uptake than baseline at higher caffeine doses. Neurotransmitters release by caffeine administration (e.g., GABA and dopamine) and the different interaction with different anesthetics could have caused the different response (28).

#### Awake Uptake Followed by Autoradiography

[^18^F]FDG uptake was compared in the conscious state and under the anesthetics ketamine + xylazine, ketamine, chloral hydrate, pentobarbital, propofol, and isoflurane (26). Although ketamine did not change the overall brain [^18^F]FDG uptake compared to the conscious animals, it did change the brain uptake pattern, with a significant decrease uptake in the frontal cerebral cortex and a significant increase uptake in the posterior cerebral cortex. The other tested anesthetics significantly reduced the tracer uptake compared to the conscious group.

In an amphetamine challenge study, it was found that the change in tracer binding potential (compared to control rats) using the dopamine D_2/3_ receptor agonist [^11^C]-(+)-PHNO compared to the change in binding potential in the same amphetamine challenge but using [^11^C]raclopride, was larger using [^11^C]-(+)-PHNO. This increased difference in BP using [^11^C]-(+)-PHNO compared to [^11^C]raclopride was observed in isoflurane-anesthetized rats, but not in conscious rats (37). This study could suggest altered dopamine levels caused by a drug/therapeutic intervention might be present in anesthetized animals but absent in clinical studies performed in conscious subjects. The authors discuss several possible mechanisms that could have caused this difference (e.g., changes in cerebral blood flow, increase dopamine release under anesthesia) but no clear hypothesis was defined.

#### Awake Radiotracer Uptake With Scan Under Anesthesia

Using the 5-HT_1A_ receptor antagonist ligand [^18^F]FPWAY, the distribution ratio of the tracer was compared in isoflurane anesthetized and conscious rats (39). Isoflurane anesthetized rats showed a significant higher hippocampus (63%) and cerebellum (32%) tracer distribution ratio than conscious rats, which the authors attribute to the decreased serotonin release in isoflurane anesthetized rats.

The influence of isoflurane and pentobarbital anesthesia on the type 1 cannabinoid receptor tracer [^18^F]MK-9470 has been investigated (40). Although absolute standardized uptake value (SUV) was not significantly different between anesthetized and awake rats, relative SUV (to the whole brain) was significantly different between anesthetized and conscious rats in several brain regions (decrease or increase). Difference was not significant when quantified using absolute SUV values, this might have been because of the higher variability in absolute SUV values compared to normalized relative SUV values.

The GABA_A_ receptor antagonist tracer [^18^F]flumazenil has been investigated under isoflurane, and ketamine/dexmedetomidine (ket/dex) anesthesia, and under the dexmedetomidine (dex) anxiolytic alone compared to awake uptake in mice (41). At 25 min post-tracer injection the frontal cortex and hippocampus uptake in isoflurane and ket/dex anesthetized mice was 10 and 3-fold higher than in awake mice. In the *ex-vivo* analysis, the hippocampus and frontal cortex uptake were significantly higher in all anesthetized conditions compared to the awake state. Either frontal cortex or hippocampus volume of distribution (calculated from pseudo-dynamic data) was significantly different under isoflurane, ket/dex, and dex compared to the awake condition. Several effects are hypothesized to have caused these differences, such as changes in cardiac output (43) and cerebral blood flow due to anesthesia (8).

In a morphine self-administration experiment, the effect of isoflurane anesthesia was investigated in the [^18^F]FDG uptake of rats that underwent withdrawal from morphine (27). Following a 45 min [^18^F]FDG uptake either awake or under isoflurane, a 30 min PET scan under isoflurane anesthesia was acquired. A significant increase in striatum glucose metabolism in the morphine withdrawal group compared to the saline group was observed after an anesthetized tracer uptake but not after awake uptake. Authors hypothesize that higher basal glucose levels in awake animals might have hindered visualizing of changes in awake animals.

#### Awake Scan in Restrained Animals

The uptake of the dopamine D_1_ receptor ligand [^11^C]SCH23390 was compared in restrained conscious rats vs. the uptake in anesthetized rats using the anesthetics chloral hydrate, ketamine, and pentobarbital anesthesia (33). Compared to the conscious state, the authors observed that the striatum binding potential of [^11^C]SCH23390 was higher using chloral hydrate and ketamine, but lower using pentobarbital. It is suggested that physiological changes (e.g., in cerebral blood flow), or blockade of the NMDA receptor by ketamine and chloral hydrate, but not pentobarbital, could have caused the difference in binding potential (33).

In another study, the binding site density (*B*_max_) and tracer affinity (1/*K*_D_) of the phosphodiesterase subtype 4 (PDE4) tracer [^11^C]-(R)-Rolipram, was compared in conscious and isoflurane anesthetized rats (42). *B*_max_ and *K*_D_ were determined from a saturation analysis. It was found that both *B*_max_ and K_D_ were significantly higher in restrained conscious rats compared to anesthetized rats. Changes in the phosphorylation status of PDE4 caused by anesthesia might have caused the differences (42).

In mice, the brain uptake of [^18^F]FDG has been investigated in awake animals and compared to isoflurane anesthetized mice (29). Brain SUV and glucose metabolic rate was significantly reduced in anesthetized mice compared to awake mice. The tracer kinetics were also modified by the use of isoflurane, indeed the [^18^F]FDG uptake plateau in awake mice was reached after about 30 min whereas with anesthetized mice the plateau was reached already after 2 min. The phosphorylation rate constant (*k*_3_) was reduced under anesthesia, but the glucose transport constant remained unchanged (*K*_1_).

Using a special restraining device in which the mouse head was restrained, but the extremities were allowed to move (free-walking state), scans using [^11^C]raclopride were compared also in whole-body restrained mice, and mice anesthetized with isoflurane (35). Heart rate in free walking mice was significantly lower than whole-body restrained mice. Striatum binding potential (calculated using simplified reference tissue model with cerebellum as reference region) was significantly lower in whole-body restrained mice and isoflurane-anesthetized mice compared with restrained free-walking mice. Although both isoflurane anesthesia and restraining stress cause increment in extracellular dopamine release, authors indicate that competition is not the main cause in binding potential differences (35).

**Figure 1:**
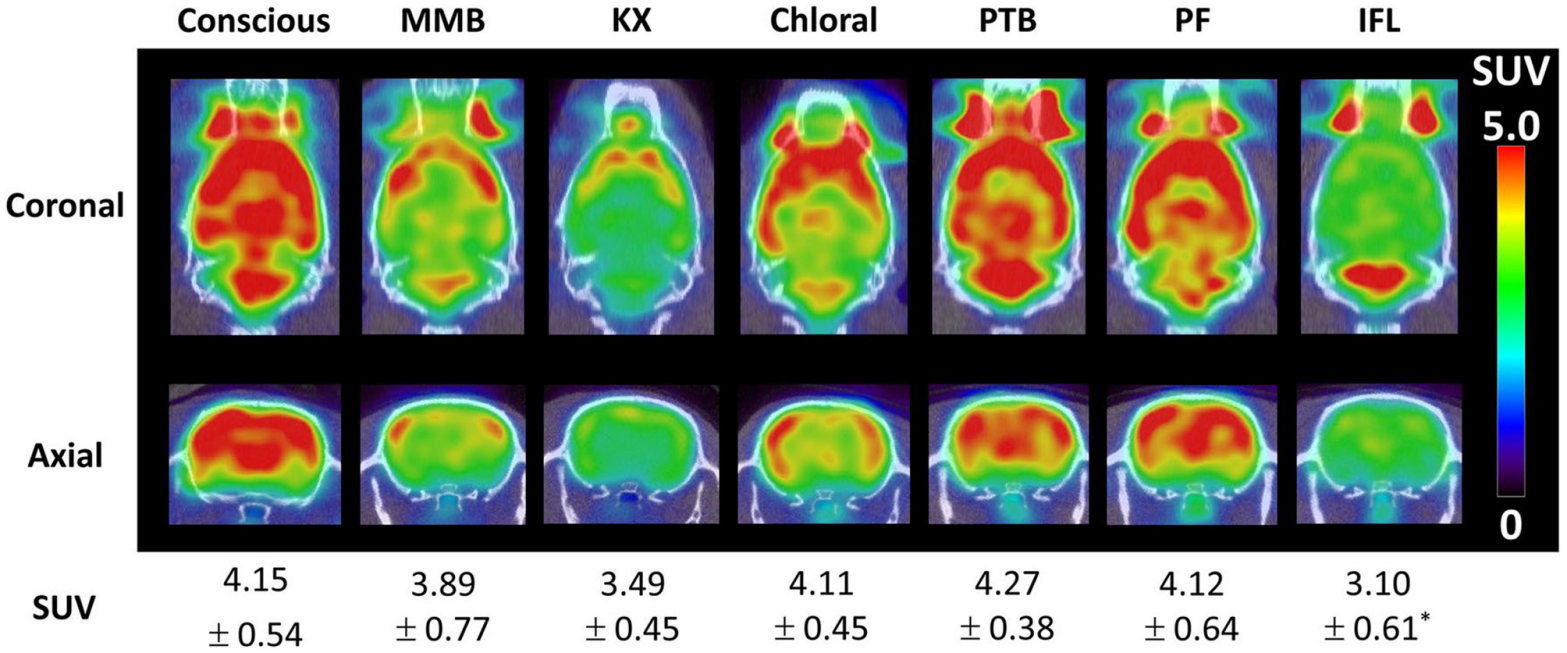
Brain [^18^F]FDG uptake in a conscious rat (using soft restrainer) and under different anesthetics (30). SUV, standardized uptake value.

Using a soft restrainer to scan rats in the awake state, [^18^F]FDG kinetic modeling has been performed and compared with scans under a mix of medetomidine, midazolam, and butorphanol (MMB) anesthesia, ketamine + xylazine, chloral-hydrate, pentobarbital, propofol, and isoflurane anesthesia (30). Two tissue compartment *K*_1_, *k*_2_, and *k*_4_ were not significantly different between conditions, but the phosphorylation reaction constant *k*_3_ was significantly lower in all anesthetic conditions compared to the awake state. The cerebral metabolic rate of glucose was significantly higher in the conscious group compared to all anesthesia groups (Figure 1). Moreover, cerebral blood flow, measure using [^125^I]IMP, was not significantly different from conscious and chloral hydrate rats, but was significantly lower (compared to conscious) for MMB, ketamine + xylazine, pentobarbital, and propofol rats, and significantly higher in isoflurane anesthetized rats.

#### Awake and Unrestrained PET Scan

The RatCAP, a miniaturized PET scanner that can be surgically attached on the rat head (36, 44), has been used to compare the [^11^C]raclopride binding potential in awake and ketamine-xylazine anesthetized rats, finding that anesthesia reduced binding potential, although not significantly.

Using motion tracking and motion correction reconstruction, the effect of isoflurane anesthesia in rat brain [^18^F]FDG uptake was investigated (31). Time-activity curves showed a faster wash-in in the brain of anesthetized rats compared to awake rats. The regional SUV in the cortex, vestibular nuclei, diencephalon, and inferior colliculi was significantly different (lower or higher) between awake and anesthetized animals.

In another study using motion tracking and motion correction, [^18^F]FDG uptake was investigated in awake and anesthetized mice (32). Allowing an awake uptake period of 20 min, mice were scanned awake freely moving and under isoflurane anesthesia. Slope of regional time activity curves was significantly different from zero (negative slope) in anesthetized mice, but not in awake mice (i.e., approximately constant uptake). This shows anesthesia can have an effect even after considering some awake uptake period (32).

### Effect of Physical Restrain in Brain PET Tomography

As described above, physical restrain is used to avoid the use of anesthesia. However, restraining stress itself represents a confounding factor also altering the uptake of the radiotracer. Sung et al. (45) performed [^18^F]FDG scans in rats that underwent restrain immobilization and awake uptake. In all conditions with restrain there was a significant [^18^F]FDG uptake difference compared to unrestrained rats in several brain regions related with stress processing (limbic system). When removing the restraining and allowing 1 h restraint free period, uptake in some of these (e.g., hypothalamus, motor cortex) regions normalized again and did not show difference compared to unrestrained rats. In some other regions (e.g., hippocampus, thalamus) this was not the case and the differences persisted. Corticosterone levels increased over time in restrained rats, plateauing at about 30 min after immobilization began, and reduced again for rats that were allowed to recover after 1 h of immobilization. These results were similar in the brain [^18^F]FDG uptake in mice (46). Brain uptake decreased in proportion to the duration of the restrain procedure (10, 20, and 40 min), with 20 and 40 min restrain showing significantly different brain uptake than in unrestrained mice.

Patel et al. (47) investigated the rat brain uptake of [^11^C]raclopride considering an awake uptake period, a methamphetamine challenge, and immobilization stress. Both restrained and methamphetamine groups showed significantly lower striatal [^11^C]raclopride binding than unrestrained rats without methamphetamine challenge, but no significant difference was found between methamphetamine and restrained rats. Alteration of neurotransmitter levels due to handling stress might have caused these differences (47).

Using reverse-phase chromatography, levels of dopamine metabolites in the nucleus accumbens septi, caudate putamen, and frontal cortex were investigated in mice undergoing restraining stress for 30 and 120 min (48). Significant increase in metabolites levels were observed in the nucleus accumbens septi after 30 min immobilization, but only after 120 min in caudate putamen and frontal cortex. This effect was likened to the effect high doses of amphetamine can have (48).

The uptake of [^11^C]-DASB, a serotonin transporter tracer, was investigated in mice exposed to chronic restraining stress (49). Mice were restrained 4h daily for 6 weeks and scanned on the 6th week with [^11^C]-DASB. Compared to control animals, significantly lower [^11^C]-DASB levels were found in the cortex of restrained animals. Given that absence of CB_1_ cannabinoid receptor activity can impair serotonin negative feedback, sensitivity of the CB_1_ cannabinoid receptor to environmental stress might have caused the effect of stress on the tracer uptake (49).

The effect of cage transport and restraining stress was investigated in the rat brain uptake of the 5-HT_1A_ receptor ligand [^18^F]MPPF (50). Hippocampal binding potential in rats undergoing cage transport, and transport plus restrain stress, was significantly higher than in control rats. Changes in 5-HT_1A_ receptor expression due to exposure to stressful situations might have caused the change in tracer binding (50).

The expression of acetylcholinesterase has also been observed to change following restrain-related stress (51). Acetylcholinesterase specific enzyme activity was significantly reduced in mice subject to 150 min immobilization.

All these evidence points that the brain response to restraining stress involves the reaction of many neurotransmitter systems (52), such as those in dopaminergic, cholinergic, and serotonergic neurons, as well as causing change of brain glucose consumption (53). Therefore, the brain response to restraining stress can interfere (e.g., by endogenous neurotransmitter competition of binding sites) with the PET reading of tracers targeting these systems, producing results that differ from unrestrained animals. Brain uptake differences comparable to those observed in drug challenges (47) can also be caused by restraining stress. Response of brain receptors to environmental stress (49), or its expression (50), can also modify uptake of tracers targeting these receptors or systems interacting with these receptors.

## Brain Pet Scans in Non-Anesthetized Unrestrained Animals to Improve Results Translation to the Clinic

From all the methods described in the previous section to study the effect of anesthetics, methods that allow scans of awake unrestrained animals resemble more closely the conditions in typical clinical PET scans. Although specialized scanners, such as the RatCAP (44), can be used to scan awake unrestrained animals, motion correction methods are more promising since typical preclinical scanners, already installed in research facilities, can potentially be used with these methods. Therefore, in the following sections we further describe the motion correction technique and the new possibilities it can bring to small animal brain PET scanning.

### Small Animal Head Motion Tracking

The motion tracking technique allows to scan awake unrestrained rodents by tracking the motion of the animal head, with any compatible tracking technology. Motion correction techniques, considering only rigid motion from the head, can then be applied to obtain motion corrected images. This method has been initially used in motion correction for clinical brain PET scans. However, motion tracking of the rodent head presents more challenges than human head motion tracking. First, the spatial resolution of preclinical scanners is usually better than in clinical scanners, with some modern systems reaching sub-millimetric resolution (54). Therefore, the tracking system should be able to deliver sub-millimetric tracking information. Second, the scanner bore in most preclinical scanners is small and narrow. If the tracking system is located on the exterior of the scanner, its field-of-measurement will be limited due to occlusion caused by the scanner bore. Third, rodent head motion can have a larger range than human head motion, and, if the scanner bore allows it, the animal can move in all directions. This is particularly detrimental for tracking systems that require the animal to be facing the tracking device. Moreover, it is necessary to track continuous motion, as opposed to discrete motion tracking which in some cases is enough for clinical PET head motion tracking (55). To overcome all these challenges, research on this technique is ongoing and several tracking methods have been proposed.

#### Characteristics of a Tracking System for Small Animal Brain PET

To use a tracking system for head motion correction in small animals, the system needs to meet certain criteria. The accuracy of the tracking system should be better than the spatial resolution of the PET scanner. If the position of a 3D point determined with the tracking system has an uncertainty larger than the spatial resolution of the scanner, the motion correction calculation will have the same uncertainty, therefore producing blurred images with respect to the image spatial resolution.

The tracking system additionally must have a high tracking frame rate in order to capture high-speed motion. For example, if the animal moves at a speed of 2 cm/s and a tracking frame rate of 30 frames per second is used, the animal would have moved about 0.7 mm within one frame, which is comparable with the spatial resolution of a preclinical PET scanner. Thus, an uncertainty of 0.7 mm will be present in the motion tracking data, which could translate into image blurring after motion correction. Is important to note that since most PET system perform reconstruction after data acquisition, is not necessary to perform real-time tracking, and therefore tracking processing can be performed off-line.

In order to minimally affect the animal due to the tracking procedure, the tracking method should be minimally invasive and with appropriate dimensions to fit with the PET scanner. This restricts the use of many tracking systems that use bulky systems or markers that cannot be attached to the animal head. Markerless tracking systems are therefore attractive for animal head motion tracking. These systems require no physical markers to track motion and instead can use, for example, either the natural features of the rodent head as reference points (see section Optical Motion Tracking Detecting Natural Head Features) or make use of projected structured light patterns on the animal head that help to calculate the 3D model of the head (see section Optical Motion Tracking Using Structured Light).

Ideally, the tracking system should also be able to track the motion of the animal irrespective of its position in the scanner field of view. However, many tracking systems have a limited field of measurement or the success of the tracking depends on the marker position itself. For example, for many optical tracking systems, the subject has to be facing the camera to be able to track its motion.

Finally, overall practicality is desired in order to be able to perform these types of scans on a regular basis. If the setup of the tracking system requires specialized personnel, laboratories lacking this type of personnel will not be able to perform the procedure. Moreover, if the setup of the tracking system or the time for the animal preparation is long, throughput can be compromised. Therefore, a practical tracking system is necessary to allow a wider spread of motion correction for preclinical brain PET scans. Below several tracking systems proposed for preclinical brain PET, with strengths and weaknesses in some of the requirements, are presented (Table 2).

**Table 2:** Characteristics of the different motion tracking techniques for small animal brain PET.

**Tracking method**	**Accuracy (mm)**	**Species**	**Frame rate (Hz)**	**Characteristics**
Optical using rigid markers	0.25	Rats	20–40	Makes use of the MicronTracker camera. Markers with a checkerboard pattern are attached on the rat head (56).
Optical using natural features	0.2	Rats	Up to 60	Several cameras obtain different views of the object. Feature detected in different views are matched to calculate their 3D location (57).
Optical using structured light	0.33	Rats	30	Makes use of the Ensenso camera. An infrared pattern projected on the object is used to calculate the object surface point cloud (58).
PET-based point source tracking	0.24	Rats/mice	Up to 60	Tracking based on the PET image. Radioactive fiducial markers are attached on the animal head, and detected in short time frames (59).

#### Optical Motion Tracking Using Rigid Markers

**Figure 2:**
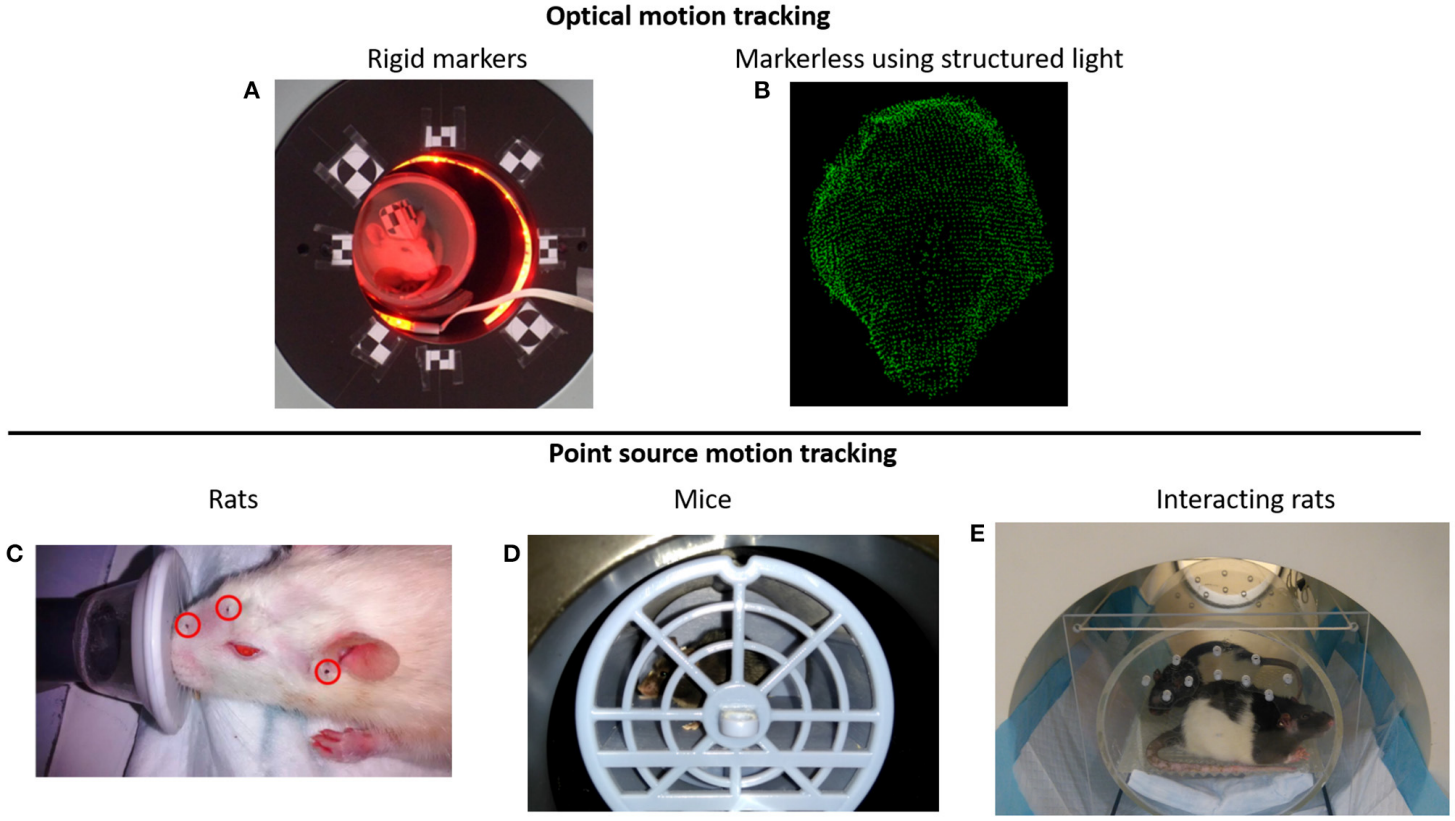
**(A)** Rat head motion tracking in the Siemens Inveon microPET scanner using the Micron Tracker device (60). **(B)** Example of a rat head point cloud calculated using structured light projection (58). **(C)** Point sources attached on the rat head for PET motion tracking (59). **(D)** Awake mouse brain PET scan using the point source tracking (32). **(E)** Awake PET scan of interacting rats performed in the HRRT scanner using the point source tracking (61).

One of the first methods proposed to track the rat's head motion makes use of the Micron Tracker camera (Claron Technology Inc., Toronto, Canada). This camera uses stereo-vision to determine the 6 degrees of freedom (3 translation coordinates and 3 rotation angles) motion of checkerboard markers that can be printed on paper and attached to a rigid surface (Figure 2A). This camera offers sub-millimetric tracking accuracy (0.25 mm), and the markers can be made small enough to be attached to the rat head. In order to synchronize the motion tracking data with the PET data, a temporal and spatial synchronization between the tracker and the PET scanner must be performed. This camera has been adapted to the Siemens Focus 220 microPET scanner (31, 62) and the Siemens Inveon microPET (60) for rat head motion tracking.

As with many optical tracking systems, the animal head must be facing the tracking camera. When the animal moves with the head opposite to the tracker, or at positions where the marker is occluded by the scanner bore, no tracking information can be acquired, and therefore the PET data during that period cannot be corrected for motion. Moreover, it might be difficult to use this marker-based camera in scanners with small bores.

This tracking system has been used together with a robotic arm in order to maximize the time the animal is inside the scanner field of view (63, 64). The platform in which the rat can move is positioned on a mechanical arm with 6 degrees of freedom motion and using the head tracking information, every time the animal moves out of the scanner field of view, it is repositioned back into the FOV.

#### Optical Motion Tracking Detecting Natural Head Features

Another approach to track the animal head motion consists of detecting natural features on the animal head to determine its 6 degrees of freedom pose (57). Therefore, no markers need to be attached to the animal head. Distinctive features that can be uniquely identified (e.g., around the eyes or nose) are determined using image feature detection algorithms (65) in images acquired from several views of the object. Then, several of these features are matched in two or more images, and the 3D position of the features can be calculated. With more than 3 of these features, the 6 degrees of freedom of the animal head can be determined. In practice, it was necessary to paint a black pattern on the animal head in order to obtain enough distinctive features to match the images in different views (57).

#### Optical Motion Tracking Using Structured Light

Combining the use of stereo vision with structured light projection the 3D surface of the animal head can be represented with point clouds (Figure 2B), which then can be used to determine the 3D pose (position and orientation) of the rat head (58). With the aid of a speckled dot pattern projected with infrared light on the surface of the object to be tracked, the 3D position of every pixel (within tracking range) in the stereo images can be determined. After acquiring the point cloud of the animal head on short time frames, the head can be registered to a reference frame using the iterative closest point algorithm (66).

#### PET-Based Motion Tracking Using Radioactive Point Sources

The use of radioactive fiducial markers to track the motion of the animal head has been proposed by our group (59). Radioactive PET point sources are attached to the rat head (Figure 2C), and by determining the spatial location of the point sources in the PET data, the pose of the head can be calculated. At least 3 non-collinear point sources are necessary to uniquely determine the pose of the animal head. Unlike the optical tracking methods presented above, this method does not require temporal or spatial calibration with the PET scanner and does not suffer from occlusion of the optical camera field of view. Therefore, is possible to track the motion of the animal in the entire scanner field of view irrespective of the animal position. Of all the tracking methods presented here, this has been the only method adapted for mice head motion tracking [Figure 2D; (32)], or for simultaneous tracking of 2 rats [Figure 2E; (61)].

### Rigid Motion Correction Reconstruction

Once the animal head motion information has been acquired with any tracking system, the PET data can be corrected for motion. Methods devised for human head motion correction can be used in small animal motion head correction (67). Event-by-event motion correction (68) has been the preferred method for small animal motion correction since methods that consider only sporadic, discrete motion (55) might perform poorly for small animal motion correction due to the erratic nature of the animal motion.

Event-by-event motion correction consists of repositioning every line of response (LOR) of the PET scan back to a reference pose using the motion tracking information. For brain motion correction only rigid motion/transformations are considered (68) with the assumption the brain only undergoes rigid motion, but non-rigid event-by-event motion correction could also be performed for respiratory motion correction (69). In theory, if one knows the pose of every individual LOR, the LOR can be repositioned individually. However, in practice, due to the finite tracking frame rate of the motion tracking system, LORs are repositioned in time bins with the same size as the tracking frame size. Interpolation between poses can be performed to calculate the pose of every individual LOR, but this has been shown to only minimally improve the image quality (56).

Once LORs are repositioned, they can be rebinned into sinograms, or reconstructed with list-mode reconstruction (68), using for example the maximum-likelihood expectation maximization algorithm (ML-EM) (70). In both cases, the LORs need to be corrected for detection efficiency (normalization). For sinogram rebinning, the compression factor of the sinogram must be considered to calculate the sinogram bin normalization factor (71). For list-mode reconstruction, the normalization correction image of all possible detectable LORs should be calculated for every motion pose. For example, for a scan time of 20 min and a tracking frequency of 30 frames per second, 36,000 normalization correction images should be calculated. This an unpractical calculation, and therefore approximations are performed to calculate normalization correction in list-mode motion correction reconstruction. One of these approximations considers only a random number of the total LORs in every pose (72), while another calculates the normalization correction image by interpolation in the image space (68). The former method has been reported to perform well-depending on the number of LORs considered and the randomization algorithm (72), while the latter performs well in terms of quantitative accuracy, comparable with motion-free reconstructions (68).

Due to the free motion of the animal in the scanner field of view, attenuation, and scatter correction need to be adapted for motion correction. Since the position of the animal body can have different orientations with respect to the head, the attenuation factors from the body also can change over time. An approximation to calculate the moving animal attenuation map considers the outline of the body activity as the attenuation map, with a constant attenuation factor of soft tissue (73). Since bone structures are small in rodents, this is a good approximation. Another solution consists on defining a “virtual scanner” fixed to the animal head to determine LORs originating in the head, and not traversing the torso (74), which then are used in the motion correction reconstruction. However, many LORs have to be discarded with this technique, degrading the image quality. Regarding scatter correction no solution has been proposed for freely moving animals motion correction, but since the proportion of scatter events in small animal brain scans is relatively small (<2.5%), this does not represent a considerable source of error (31).

Additional corrections can be performed to improve the image quality of the motion-corrected reconstruction. One of these corrections estimates the blurring caused by the uncertainty in the motion data to calculate a deconvolution correction in the motion-corrected image (60). Another correction calculates the motion-dependent point spread function of the motion scan by attaching a point source to the moving subject (75). The point spread function calculated from the point source image is then used in a deconvolution correction. Correction for the parallax effect in motion scans has also been developed (76). For this method, the motion-dependent and spatially variant point spread function of the motion scan is analytically calculated for every voxel in the image and then used for resolution modeling in the ML-EM reconstruction.

### Novel Possibilities for Brain Behavioral Studies in Freely Moving Awake Rodents

Although one of the main motivations to perform motion correction PET scans of awake animals is to circumvent the use of anesthesia, this scanning setup also allows to perform new experimental designs not possible in anesthetized or restrained animals. In particular, quantification of the animal behavior during the PET scan has been explored using motion correction.

**Figure 3:**
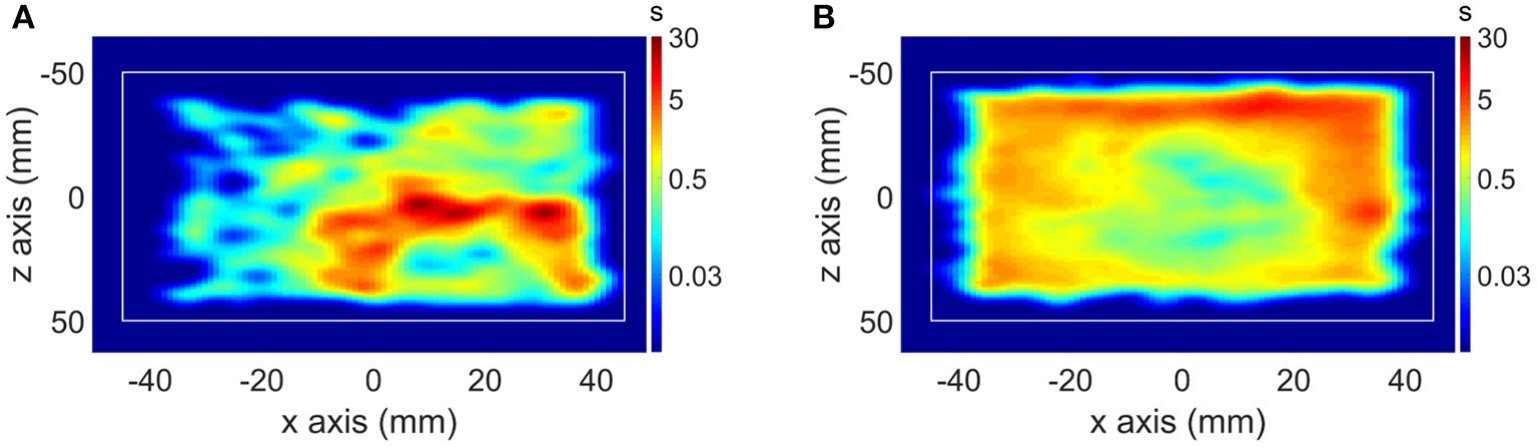
**(A)** Motion histogram (top view) of a mouse PET study in a control scan and **(B)** motion histogram of the same mouse in a memantine challenge scan.

One of the first studies in which the animal behavior was quantified simultaneously during the PET scan was in mouse memantine challenge scans performed with the point source tracking (32). The mouse behavior was observed during the PET scan in a memantine challenge experiment, showing significantly increased locomotion in memantine challenge mice compared to control mice (Figure 3). Another novel experimental setup using the point source tracking is the scan of freely moving and interacting rats (61). The large field of view of the human brain High Resolution Research Tomograph allowed tracking of 2 freely moving rats placed in a cage fitted to the FOV of the scanner.

Using marker-based motion tracking, Kyme et al. (64) investigated the effect of an amphetamine challenge in the binding of [^11^C]raclopride. Using rats with an implanted catheter, [^11^C]raclopride was administered in the awake state, followed by administration of amphetamine 20 min later. After amphetamine administration [^11^C]raclopride binding significantly reduced compared to saline administered rats, while locomotion increased.

## Discussion

The effect of anesthesia in small animal brain PET has been demonstrated in studies performed since the early days of microPET. Since studies investigating the mechanisms of action of anesthetics demonstrate that they modify physiological parameters (e.g., respiratory rate, cerebral blood flow, etc.) and interact with neurotransmitter systems, it is expected that anesthetics will also modify the brain response to PET tracers. Tracers targeting receptors such as dopamine, serotonin, and GABA_A_, as well as the glucose analog [^18^F]FDG, have been demonstrated to be influenced by anesthetics during PET tomography.

Among the suggested mechanisms causing the difference in tracer binding due to anesthesia are the changes in physiological parameters. Changes in cerebral blood flow caused by anesthesia has been indicated as one of the possible factors producing differences in the tracer uptake compared to the awake state (34, 47). For dopamine receptor tracers, although competition with the dopamine release caused by the anesthetic (e.g., isoflurane) can be linked to changes in the tracer uptake, this competition is assumed to be minimal due to the low level of dopamine release caused by the anesthetic (37, 39). Similarly, inhibition of serotonin release due to isoflurane has been suggested as a possible cause on the different uptake of the serotonin receptor 5-HT_1A_ tracer [^18^F]FPWAY compared to the awake state, although this was not proven (39).

Due to the methodological differences of the studies presented, different additional confounding factors can be present, making a direct comparison of the reported results difficult. For example, studies allowing awake uptake followed by anesthesia scanning might have already an influence of anesthesia, especially for reversible tracers, depending on the pharmacokinetics of the anesthesia. In addition, stress caused by the different handling procedures, even in freely moving animal scans, can have an influence in the PET outcome. However, considering all the studies presented with different possible confounding factors, it is clear that anesthesia can influence the radiotracer uptake and kinetics in small animal brain studies.

Among the different methods used to elucidate the effect of anesthesia on the animal brain PET reading, motion tracking together with motion correction reconstruction most closely resemble the condition in the clinic, i.e., awake and unrestrained. Overall, the tracking systems presented here have good enough tracking accuracy (~0.25 mm) relative to the spatial resolution of typical preclinical scanners (1.5–1 mm). In addition, although motion correction reconstruction methods require approximations, images have comparable quality and quantitative accuracy to motion-free reconstructions.

As demonstrated in the mouse memantine (32), and rat amphetamine (64) challenge studies, the behavior of the animals in response to a drug can also be observed and quantified simultaneously with the PET scan using motion correction. Response to other non-pharmacological challenges could also be investigated in freely moving animals using PET, such as response to visual (77), olfactory (78), and auditory stimulus (79). Finally, more elaborated experiments, involving for example interaction of animals (61), or the response of the animal to a training or conditioning/reinforcement experiment, could be explored in freely moving animals with PET.

One of the main reasons motion tracking and motion correction for brain PET scans is not widely adopted is the need for complex software, and sometimes additional hardware, to perform these procedures. Improving practicality and ease of use would allow the adoption of this technique for regular implementation in PET brain small animal scans. Therefore, additional collaboration between industry and research centers would be needed for the wider adoption and implementation of these types of scans.

## Conclusions

The use of anesthesia in small animal preclinical brain PET studies represents the main confounding factor for proper translational understanding of animal models. Since clinical PET studies are usually performed without the use of anesthesia, translation of results from anesthetized animals to awake patients can also be compromised. Motion tracking with subsequent motion correction reconstruction offers the possibility to perform brain PET scans in freely moving unanesthetized animals. Several research developments have been performed in the last years, improving the accuracy and practicality of this technique, allowing the simultaneous study of animal behavior and molecular brain PET imaging. Further research and collaboration with the industry would allow wider adaptation of motion tracking and motion correction for brain PET preclinical scans.

## Author Contributions

AM, DB, SStr, SSta, and JV were involved in drafting and editing the manuscript and figures. All authors approved the final manuscript and they are accountable for the content of the work.

## Funding

AM was supported by the ERA-NET NEURON project SleepLess supported by BMBF (01EW1808) and FWO under the frame of Neuron Co-fund, and by a Research Project (G0A8517N) from the Research Foundation Flanders (FWO). DB was supported by the Research Foundation Flanders (FWO) through a post-doctoral fellowship (1229721N). The University of Antwerp also founded the work through a partial assistant professor position for JV and a full professor position for SSta.

## Conflict of Interest

The authors declare that the research was conducted in the absence of any commercial or financial relationships that could be construed as a potential conflict of interest.

## Publisher's Note

All claims expressed in this article are solely those of the authors and do not necessarily represent those of their affiliated organizations, or those of the publisher, the editors and the reviewers. Any product that may be evaluated in this article, or claim that may be made by its manufacturer, is not guaranteed or endorsed by the publisher.
